# Serological detection of avian reovirus in different poultry flocks of Gazipur and Mymensingh districts of Bangladesh

**DOI:** 10.14202/vetworld.2019.1126-1131

**Published:** 2019-07-26

**Authors:** Syeda Farjana Neepa, Zobayda Farzana Haque, Abdullah Al Momen Sabuj, Md Alimul Islam, Sukumar Saha

**Affiliations:** Department of Microbiology and Hygiene, Faculty of Veterinary Science, Bangladesh Agricultural University, Mymensingh-2202, Bangladesh

**Keywords:** avian reovirus, enzyme-linked immunosorbent assay, seropositive

## Abstract

**Background and Aim:**

Avian reovirus (ARV) is a constraint to poultry industry in Bangladesh as a cause of several diseases in chickens, especially in broiler. However, the actual status of the viral infection is not known because the large-scale study is not conducted in this country. Therefore, this study aimed to check the presence and distribution of ARV-specific antibody in respect to area, types of chickens (broiler breeder, broiler, and layer), vaccination status, and age of chickens in Gazipur and Mymensingh districts of Bangladesh.

**Materials and Methods:**

A total of 276 chickens’ blood samples were collected from two well-organized broiler breeder stock, seven broiler farms, and five layer farms located at two districts, namely Gazipur and Mymensingh of Bangladesh. Blood samples were collected from wing vein of the apparently healthy chickens using 3 ml of syringe and serum was harvested by keeping the syringe at room temperature in slanting position. The sera were transferred to the laboratory by maintaining the cool chain and further processing was performed by indirect enzyme-linked immunosorbent assay using ARV antibody test kit.

**Results:**

The results of serological test revealed that an average of 39.5% seropositive against ARV was recorded in chickens of Gazipur and Mymensingh districts. Among these, chickens of Gazipur district had the highest seropositivity of 50.5% than Mymensingh (30.7%). With respect to vaccination status, the seropositivity of vaccinated chickens in both areas was 100% and non-vaccinated chickens was 50.5% in Gazipur and 30.7% in Mymensingh district, respectively. However, regarding age groups, the seropositivity was higher in the age of 4-6 weeks (64.5%).

**Conclusion:**

The present serological findings showed a higher prevalence of ARV-specific antibodies in broiler birds. It indicates that the poultry industries of Bangladesh are contaminated with ARV which may naturally be transmitted to chickens either vertically or horizontally.

## Introduction

Bangladesh is a country having a growing population with an increasing demand of protein. To meet up this increasing demand of protein for the added people per year, the poultry sector is trying to contribute through increasing the meat and egg production. Although poultry meat provides approximately 38% of total animal meat in the country [1], most of the people are still living at a low nutritional status. The progress in poultry farming and production is being hampered in Bangladesh due to some of the constraints. One among them is the outbreak of some diseases causing higher mortality and stunting the growth performance of chickens. This may be due to some prevalent and recurrent diseases of both infectious and non-infectious type. This occurrence is not only reducing the protein supplement for the people of the country but also causing financial loss to the growing poultry industry.

Avian reovirus (ARV) infections of chickens are major causes of economic problems for commercial poultry producers throughout the world [2]. Economic losses caused by ARV infections in chickens are often the result of poor performances such as extenuated weight gains, high feed conversion ratio, decreased egg production, reduced marketability of affected birds, and also increased diseases susceptibility [3]. It belongs to the *Orthoreovirus* genus in the *Reoviridae* family [4]. Structurally, it is non-enveloped, icosahedral virion with 75-80 nm in size and double-stranded RNA genome having 10-12 segments [5].

Poultry species such as chickens and turkey are prone to ARVs infection for their heterogeneous pathogenicity [6]. In chickens, the most recognized form of ARV-associated diseases and also a significant cause of lameness is infectious tenosynovitis or viral arthritis [7]. Depending on the degree of severity of the inflammation, an affected bird may be unable to move toward feed and water resulting in poor growth or death [8]. In broilers, other significant symptoms are stunting, malabsorption syndrome (MAS), growth retardation, pericarditis, myocarditis, and enteritis [9]. Immunosuppression is also a subsequent result of ARV infection in chickens [10] which may predispose them for other stress factors and infectious agents in environment. Immunosuppression by this virus may also retard the success rate of vaccination against other infectious diseases such as infectious bursal disease and hepatitis [11]. ARV can be transmitted both vertically and horizontally [12]. Although ARV are not always pathogenic and have been reported from routine examination in apparently healthy poultry flocks [13]. However, chickens are more susceptible to ARV in the immediate post-hatching period and become increasingly resistant to infection with age [14]. Morbidity is variable can be reached up to 100% and mortality is low generally <6% in case of this viral infection [13].

For vaccination to have amenities of success, it must have to protect the birds at their early age. Attempts to check the infection in chickens have guided to the establishment of a number of ARV vaccines, both live and inactivated. To develop a vaccine, the present status of this virus among the poultry farms of particular area should be evaluated. Enzyme-linked immunosorbent assay (ELISA) is a pretty good sensitive test that is very convenient to use in examining large numbers of sera and its commercial kits are available [15]. Intensive commerce with poultry material contributed to the spread of the infections with ARV, and after 1990, these infectious diseases frequently evolving all over the world [16]. Despite the country with a large number of poultry farms, only few evidence of incidence of ARV has been reported in Bangladesh. ARV was for the 1^st^ time reported in June 1997 in Animal Research Division, Bangladesh Livestock Research Institute [17]. After that, another study was carried out in Dinajpur district of Bangladesh by Salam *et al*. [3] on ARV antibodies in layer birds of small-scale commercial farms.

The present study was undertaken to perform a serological survey to check the presence of ARV antibodies in chickens as well as to determine the distribution of its specific antibody in respect of the area, vaccination, types of bird (broiler breeder, broiler, and layer), and age of birds of Gazipur and Mymensingh districts of Bangladesh.

## Materials and Methods

### Ethical approval

Experiments involving animals were reviewed and approved by the Animal Welfare and Experimentation Ethics Committee of Bangladesh Agricultural University, Mymensingh, Bangladesh (ref no. AWEEC/BAU/2018(15)).

### Source of samples

A total of 276 blood samples were collected from broiler breeder, broiler, and layer chickens located at two districts, namely Gazipur and Mymensingh of Bangladesh. During the sample collection, the area, type of birds (broiler breeder, broiler, and layer), vaccination status, and age of birds were considered. Of 276 samples, 28 were collected from two broiler breeder stocks, 152 from seven broiler farms, and 96 from five layers farms of selected district areas, respectively. The broiler breeders were vaccinated against ARV and used as a positive control in this experiment and others were not.

### Collection of blood, transportation, and serum preparation

Blood from wing vein of randomly selected apparently healthy chickens were collected using 3 ml of syringe and serum was harvested by decanting as described by Amini *et al*. [18]. After the blood clotted within the syringes, the blood samples were kept in icebox and transported to laboratory by maintaining cool chain. Then, sera were subjected to spin at 3000 rpm for 5 min to remove the remaining clots, red blood cells, and other insoluble materials and stored at −20°C for performing the indirect ELISA.

### ELISA test

The collected and processed sera samples were analyzed by indirect ELISA. ELISA was conducted using ARV antibody test kit (ID Screen^®^ ARV Indirect, ID. Vet, Grabels, France) containing ARV antigen-coated plates. In case of ELISA, samples were tested at a final dilution of 1:500 in dilution buffer where it was first prediluted at 1:50 dilution, followed by 1:10 dilution. Then, 100 µl of negative control was added into A1 and B1 wells of ARV-coated plate and positive control was also added into C1 and D2 wells. Remaining 92 wells were filled individually with 100 µl of diluted serum samples and the plate was covered with lid and incubated for 30 min at 21°C (±5°C) at dark condition. Meantime 1× conjugate was prepared by diluting the concentrated conjugated 10× to 1:10 in dilution buffer. After incubation, the wells were emptied and washed each well 3 times with approximately 300 µl of the wash solution 1×. Then, 100 µl of the prepared conjugate was added into each well. Again, the plate was covered with lid and incubated for 30 min at 21°C (±5°C). After that, the plate was emptied and washed with wash solution and 100 µl substrate solutions were added into each well. The plate was incubated for 15 min at 21°C (±5°C). Then, the reaction was stopped by adding 100 µl stop solution. Finally, the optical density value of each sample was measured at 450 nm and recorded for calculating sample to positive (S/P) ratio and antibody titer.

### Interpretation of result

For each sample, S/P ratio and antibody titer were calculated using the following formulas:

For S/P ratio:


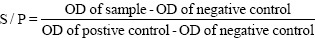


For antibody titer: Log_10_(titer)=1.1×log_10_ (S/P) +3.700; titer=10^log^_10_(titer)

Results were interpreted as follows:

**Table:** 

S/p-value	ELISA antibody titer	Reovirus immune status
S/≤0.2	Titer≤854	Negative
S/≥0.2	Titer>854	Positive

ELISA=Enzyme-linked immunosorbent assay

## Results

### Serological detection of ARV according to areas

The overall seropositive of ARV was found 39.5% in both Gazipur and Mymensingh district, of which highest one of 50.5% was observed in Gazipur district followed by Mymensingh (30.7%) (Table-1).

**Table 1:** Serological detection of ARV in different experimental areas and types of chickens.

District (experimental areas)	Types of birds	Number of sera tested	Number of positive sera	Seropositive (%)	Overall seropositivity according to district (%)	95% CI			Average of seropositive (%)	95% CI		
	
						SE	L (%)	U (%)		SE	L (%)	U (%)
Gazipur	Broiler breeder*	14	14	100.0	50.5	±9.3	41.2	59.8	39.5	±6.08	33.42	45.58
	Broiler	72	56	77.8								
	Layer	39	0	0.0								
Mymensingh	Broiler breeder*	14	14	100.0	30.7	±7.7	23	38.4				
	Broiler	80	42	52.5								
	Layer	57	0	0.0								

L=Lower limit, U=Upper limit, SE=Mean standard error, Broiler breeder

* =The seropositive of broiler breeder is used as positive control in this research and this result is not included in overall seroprevalence according to district (%) and average seroprevalence (%) study, ARV=Avian reovirus, CI=Confident interval

### Serological detection of ARV according to the types of chickens

Three types of chickens including broiler breeder, broiler, and layer were selected for the investigation of seropositive against ARV. In broiler breeder, 28 (100%) samples of 28 were seropositive. In broiler, 98 (64.5%) samples of 152 were seropositive, whereas no layer samples were found to be seropositive for ARV (Table-1). The seropositive among broiler breeder in two districts was 100% and broiler birds in two districts was as follows, Gazipur (77.8%) and Mymensingh (52.5%).

### Serological detection of ARV according to vaccination status

Of 276 chickens, 28 chickens were vaccinated against ARV with modified live vaccine through drinking water which were broiler breeder (positive control) and other 248 were non-vaccinated which were broiler and layer birds. The seropositive among vaccinated chicken in Gazipur and Mymensingh was 100% and non-vaccinated chicken in Gazipur and Mymensingh was as follows 50.5% and 30.7% (Table-2). The coefficient of variation (CV) was recorded higher in non-vaccinated than vaccinated chickens.

**Table 2:** Serological detection of ARV according to the vaccination of chickens.

District	Vaccination condition	Number of positive samples/total tested samples	Seropositive (%)	Antibody titer mean	Antibody titer (SD)	Antibody titer (CV)
Gazipur	Vaccinated	14/14	100.0	5875	410.8	7.0
	Non-vaccinated	56/111	50.5	1979.1	218.7	11.1
Mymensingh	Vaccinated	14/14	100.0	7135	490.4	6.9
	Non-vaccinated	42/137	30.7	1404.7	178.7	12.7

SD=Standard deviation, CV=Coefficient of variation, ARV=Avian reovirus

### Serological detection of ARV according to farm

Table-3 shows that all 28 samples of broiler breeder from two farms were found positive for specific ARV antibodies. The ELISA result revealed the presence of specific ARV antibodies in the entire broiler tested. Of 152 samples from a total of seven farms, 98 (64.5%) were found positive while only 54 (35.5%) were observed to be negative. At the farm level, 3 farms (42.86%) had 100% positive results, while other birds from four different farms were 20.0%, 31.6%, 5.6%, and 68.0% positive for specific ARV antibodies. Among seven farms, the farm F showed the lowest CV was 4.8. In layer bird, all 96 samples from five farms were found negative for ARV antibodies (Table-4).

**Table 3:** Serological detection of ARV in broiler according to farm.

District	Farm	Number of positive samples/total tested samples	Seropositive (%)	Antibody titer mean	Antibody titer (SD)	Antibody titer (CV)
Gazipur	A	36/36	100.0	3611.3	212.4	5.9
	B	16/16	100.0	2778.2	303.1	10.9
	C	4/20	20.0	1171.5	317.9	27.1
Mymensingh	D	6/19	31.6	955.3	296.1	31.0
	E	1/18	5.6	746.2	202.5	27.1
	F	18/18	100.0	4725.3	227.8	4.8
	G	17/25	68.0	4294.3	531.7	12.4

SD=Standard deviation, CV=Coefficient of variation, ARV=Avian reovirus

**Table 4:** Serological detection of ARV in layer according to farm.

District	Farm	Number of positive samples/total tested samples	Seropositive (%)	Antibody titer mean	Antibody titer (SD)	Antibody titer (CV)
Gazipur	A	0/39	0.0	355.5	41.4	11.7
Mymensingh	B	0/16	0.0	42.4	15.5	36.5
	C	0/16	0.0	184.7	40.2	21.8
	D	0/16	0.0	163.9	53.6	32.7
	E	0/9	0.0	125.8	61.8	49.2

SD=Standard deviation, CV=Coefficient of variation, ARV=Avian reovirus

### Serological detection of ARV according to the age groups

The collected sera samples were grouped into three categories based on age of the chickens such as 4-6, 60-70, and 71-81 weeks which were examined for the presence of ARV antibodies (Table-5). In the age group of 4-6 weeks, of 152 samples were subjected to test, 98 (64.5%) were seropositive. No samples were observed for seropositive against ARV in the age groups of 60-71 and 70-81 weeks.

**Table 5:** Serological detection of ARV according to the age groups of chickens.

Age group (weeks)	Number of positive samples/total tested samples	Seropositive (%)	Antibody titer mean	Antibody titer (SD)	Antibody titer (CV)
4-6 (broiler bird)	98/152	64.5	2611.7	298.8	11.4
60-70 (layer bird)	0/48	0.0	240.5	51.6	21.5
71-81 (layer bird)	0/48	0.0	130.3	36.4	27.9

SD=Standard deviation, CV=Coefficient of variation, ARV=Avian reovirus

## Discussion

ARV has been reported in many poultry farms in different parts of the world in recent past with chickens suffering from various symptoms such as stunting growth, MAS, enteritis, respiratory symptoms, pericarditis, and myocarditis [13]. The disease is economically important to poultry industry of Bangladesh because lots of people directly or indirectly dependent on this sector [3]. However, the study on ARV in chickens of Bangladesh has been conducted in the past; the reports are very less. Considering the large poultry population in Bangladesh, the present study was conducted. Under this study, serological detection of ARV infections in broiler breeder, broiler, and layer chickens in Gazipur and Mymensingh district of Bangladesh was investigated using indirect ELISA. Serum samples (n=276) collected from chickens in two broiler breeder, seven broiler, and five layer farms were tested for antibodies against ARV. Among them, 39.5% of serum samples were found seropositive. All the broiler breeder samples were 100% seropositive, whereas most of the broiler farm chickens showed seropositive for ARV. Rates of seropositivity (77.8%) were higher in broiler chickens of Gazipur district than the broilers of Mymensingh (52.5%). Overall, seropositivity was higher in Gazipur district than Mymensingh (50.5% vs. 30.7%). In Ontario, similar study was carried out by Nham *et al*. [19] who recorded 91% of seroprevalence of ARV in broiler, which was higher than the present study. In China, 92% of unvaccinated chickens showed positive for ARV infection [20]. In another study conducted in Saskatchewan, Canada, reported that 98.3% of samples were seropositive for ARV-infected samples [21]. Baksi *et al*. [22] reported lower seroprevalence (8%) of ARV infection in different parts of India. In Bangladesh, a study was carried out by Biswas *et al*. [23] in chickens on smallholdings reported lower seropositivity of 47% (138/295) than the current study (64.5%). Another study conducted in Dinajpur district of Bangladesh by Salam *et al*. [3] reported 93.33% ARV seroprevalence in layer chickens that are completely opposite to the present study. While we cannot clearly explain the unexpected findings of Salam *et al*. [3], they may reflect differences in the study area, biosecurity measures of the individual farms, but most likely sampling from small number of farms in our study may not provide the actual picture of reovirus infection in layer farms of Bangladesh. This area needs to be investigated further for a better conclusion of the reovirus scenario in Bangladesh. These data suggest that chickens of Gazipur district have higher infection than Mymensingh district, especially in broiler chicken as most of the poultry sector cover with broiler chickens. Between the two districts, Gazipur is well known for poultry rearing with higher density of farm and this could be another cause of higher seropositivity of ARV infection in this district compared to Mymensingh.

According to vaccination status, the highest seropositivity was recorded in vaccinated chicken over non-vaccinated. All the samples of broiler breeder were observed seropositive for ARV infection as it was vaccinated. Antibody titers recorded in broiler breeder in two study areas were found higher than the broiler. It might be happen because long-term antibody is developed as a result of constant reinfection of virus subclinically in broiler breeder [13]. Erol and Sengul [24] also observed higher antibody titer in broiler breeder against ARV in Turkey. Seropositivity of ARV was higher in the age of 4-6 weeks than other two age groups. It is possible because immune system of young chickens is not fully developed, whereas older chickens developed age-related resistance [25].

In summary, the presence of ARV infections in the study areas is significantly higher that may cause severe economic loss in poultry sector. Broiler breeder stock is highly infected with ARV, but the presence of virus is also prominent in broiler. In broiler, it causes arthritis/tenosynovitis including the sign of swelling and inflammation of joint may lead to paralysis. Sometimes, it can cause serious respiratory problem. Data on seroprevalence of ARV infections in Bangladesh are limited. Few researches have been conducted previously and there are no recent and adequate data available in Bangladesh. Therefore, losses caused by ARV infection in chickens are still unknown to poultry farmer. To the best of our knowledge, this is the first study conducted on broiler breeder, broiler, and layer chickens together regarding ARV infection in two districts using indirect ELISA. Therefore, further research should be focused on virological and molecular area to combat the ARV infections in Bangladesh.

## Conclusion

The magnitude of the distribution of the presence of ARV antibodies revealed that broiler birds from 4-6 weeks are susceptible to ARV. Furthermore, there is a good distribution of this virus among farms of different regions which reveals that pathogenicity of the ARV may be influenced by some other factors. More study should be performed to identify the potential risk factors and more precise status of this virus in the large-scale farms of this country. Furthermore, awareness should be promoted among all category of farmers to minimize the risk and economic loss in commercial poultry industry relevant to ARV infections.

## Authors’ Contributions

SFN, ZFH, and AAS carried out the experiments, analyzed the data, and wrote the initial draft of the manuscript. SS and MAI designed and supervised research work and finalized the manuscript.
